# A checklist of Mantodea of Belize, with a regional key to species

**DOI:** 10.3897/zookeys.1068.58193

**Published:** 2021-11-04

**Authors:** Lohitashwa Garikipati, Jason E. Bond

**Affiliations:** 1 Department of Entomology and Nematology, University of California Davis, 1 Shields Avenue, Davis, CA, USA University of California Davis Davis United States of America

**Keywords:** Dictyoptera, key, Lucid interactive key, mantises, microhabitat, neotropical

## Abstract

The list of known Mantodea in Belize is updated, with notes of 12 new country records, bringing the total known species of Mantodea in Belize to 21. Further information on habitat and microhabitat observations are included. A regional dichotomous key and Lucid interactive key are provided to known species in Belize. A list of other possibly endemic species is provided. Remarks on the merit of further sampling efforts in central America are made, based on recent studies. Our findings suggest that our understanding of Central American Mantodean diversity could be vastly improved by further documentation.

## Introduction

Mantodea, despite being a relatively well-known insect group to the public, has historically received little attention ecologically and taxonomically. Prior to Svenson and Whiting (2004), all the work done on the systematics of Mantodea were based on morphological characters. The study by Svenson and Whiting in 2004 and further (more comprehensively) in 2009, highlighted the rampant paraphyly across the order due to homoplasic traits previously thought to be synapomorphic (Svenson and Whiting 2004, 2009). Recent work by Rivera and Svenson (2016) has significantly improved our current understanding of the order’s systematics with the establishment of the Acanthopoidea, the polymorphic earless mantises, along with further clarification of relationships among the various Neotropical mantodean groups. This groundbreaking work has been advanced by Schwarz and Roy (2019), who altered the familial and subfamilial relationships of the Artimantodea. Phylogenetic work by Svenson et al. (2016) resolved the subfamilial relationships of the neotropical Vatinae and Stagmomantinae. This was supported by Rodrigues et al. (2017) who generated a phylogeny with strong monophyletic support in the Stagmomantinae and included the description of *Hondurantemnachespiritoi*, a large mantid from the montane regions of Honduras and southeastern Mexico.

The recent discovery of large Mantodea like *H.chespiritoi* Rodrigues et al. suggests that there are still species, even large ones, that remain undiscovered (Rodrigues et al. 2017). Thespidae are a family of largely understudied, cryptic mantises that primarily mimic sticks and twigs, although some, such as *Thesprotiella* Giglio-Tos and *Pseudopogonogaster* Beier, mimic mosses and lichen (Rivera and Svenson 2016). Moulin and Roy (2020) highlighted the vast genetic divergence from seemingly morphological conspecifics, indicating that Mantodea is likely more speciose than previously expected. With so much work remaining to be done systematically, further sampling efforts are imperative to ensure as many taxa as possible have been examined and catalogued.

The mantodean diversity of Belize is not well known. Most of our understanding of Belizean fauna comes from documentation by Saussure and Zehntner in the late 19^th^ century (Saussure 1861, 1870, 1871; Saussure and Zehntner 1894). A few species were documented later by other authors such as *Mellieramordax* (Rehn 1935). However, to our knowledge, the addition of Belizean mantodean fauna was minimal, with Rivera (2010) noting seven species from the country. Numerous efforts have been conducted in other Neotropical countries to document mantodean diversity, primarily in regions in the Amazon rainforest (e.g., Peru, Ecuador, Brazil, and Colombia), northern South America, Mexico, and Costa Rica (e.g., Battison et al. 2005; Battison and Picciau 2008; Rivera 2010b; Rivera et al. 2017). As the discovery of *Hondurantemna* Rodrigues et al. indicates, there is still more work to be done in central America. Currently, several countries remain poorly sampled for mantodean diversity, with Belize previously having only nine recorded species (Rivera 2010; Maxwell 2014). Here we note 12 more species that represent, to our knowledge, new country records. We provide regional dichotomous and Lucid interactive keys to the species known in Belize, and we include observational details on microhabitat preferences of the specimens we collected. Based on records from surrounding countries, we also predict other species that are likely in Belize but have yet to be sampled.

## Materials and methods

**Figure 1.**
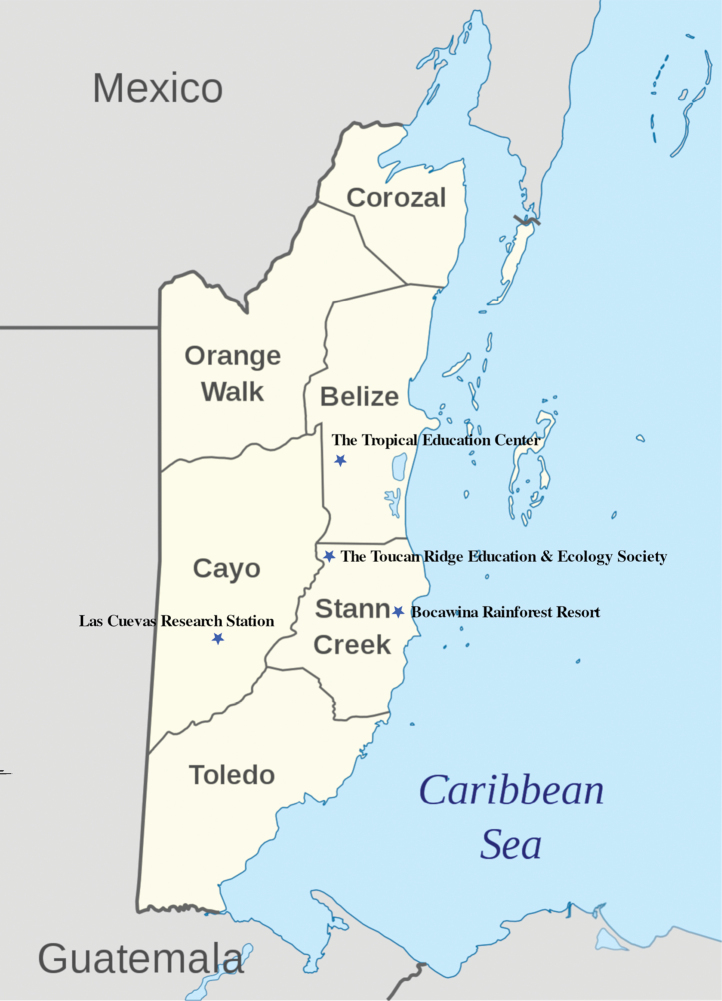
Collection locations of the 2019 BioBlitz.

All Mantodea were collected in a two-week bio blitz with the Bohart Museum of Entomology. The bio blitz spanned four locations (see Fig. 1) in Belize, from 22 June to 6 July 2019. Three nights were spent at each location, and a total of 16 collectors (including the authors) collected various groups of arthropods.

Specimens were photographed in situ when possible, captured, and subsequently killed using an ethyl acetate kill jar. Collecting typically occurred from 9 am to 2 am. Insects were located by sight and collected by hand, in light traps, or in soap traps. Observational microhabitat data was noted when possible for specimens in situ. Preservation and nomenclature follow Brannoch et al. (2017). Systematics follows Schwarz and Roy (2019) for the Cernomantodea, and Rivera and Svenson (2020) for the Acanthopoidea. Specimens were either placed into 95% EtOH or pinned soon after collection. Genitalia were extracted in heated 10% KOH and subsequently preserved (as in Brannoch et al. 2017) for identification and imaging. Photographs taken in the field were taken with an Olympus TG-5 Camera. Figures and diagrams were edited and finalized in GIMP.

Images for the Lucid interactive key were taken via Leica Application Suite and edited in Photoshop. Characters were scored using a Leica M205 C microscope.

## Results

We collected over 50 specimens as part of this survey, spanning 14 genera and 19 species. Previously, there were nine recorded species from Belize (Ehrmann 2002; Rivera 2010; Maxwell 2014; Patel and Singh 2016a, 2016b; Patel et al. 2016). We have added 12 more species to this list, bringing the total known number of Belizean species to 21. A few taxa, such as *Thesprotia* Stål, have vague descriptions, and this makes it difficult to identify the nymphs we collected. Morphological characters are still unstable, as thespids undergo significant morphological changes in their post-embryonic development (Svenson and Rivera 2016). We also gathered preliminary information on the microhabitat preference of the mantodean fauna collected, which we note below.

Using the specimens collected we generated a dichotomous key (below) and a Lucid interactive key (http://www.lucidcentral.org). This Lucid interactive key is available at https://keys.lucidcentral.org/keys/v4/mantodea_of_belize/. The aim is to provide both citizens and entomologists unfamiliar with this group with an easy method to identify collected species with high accuracy. Photographs in the Lucid key are included to aid with character determination.

### Checklist of the Mantodea of Belize

The following abbreviations are used for the districts of Belize:

**CO** Corazal;

**OW** Orange Walk;

**BD** Belize District;

**CD** Cayo District;

**SC** Stann Creek;

**TD** Toledo District.

The following abbreviations will be used for locations in Belize (all locations in Fig. 1 below):

**T.E.C.** Tropical Education Center;

**T.R.E.E.S. Field Station** Toucan Ridge Education and Ecology Society Field Station.

#### FAMILY MANTOIDIDAE

***Mantoidamaya* Saussure & Zehntner, 1894 (New country record) [CD, BD**]

**Type locality.** Mexico: North Yucatan, Temax.

**Material examined.** BZE: Belize Dist. 3 ♂, 2 ♀, collected by light trap in the vicinity of Tropical Education Center., BZE: Cayo Dist: 5 ♂, collected in same method as above 3 ♂ collected by hand on branches, in the vicinity of Las Cuevas Research Station.

#### FAMILY THESPIDAE Saussure, 1869

##### SUBFAMILY PSEUDOMIOPTERYGINAE Giglio-Tos, 1915

***Pseudomiopteryxinfuscata* Saussure & Zehntner, 1894 (New country record) [BD, CD, SC**]

**Type locality.** Panama: Volcan de Chiriquí.

**Material examined.** BZE: Belize Dist – 1 ♀ collected by hand in the vicinity of Tropical Education Center; BZE: Stann Creek Dist: 1 ♂ collected by hand in the vicinity of T.R.E.E.S. Field Station; BZE: Cayo Dist: 1 ♂ collected at light trap in the vicinity of Las Cuevas Research Station; BZE: Stann Creek Dist: 5 ♂, 1 ♀ collected by soap trap in the vicinity of Bocawina Rainforest Resort.

**Figure 2.**
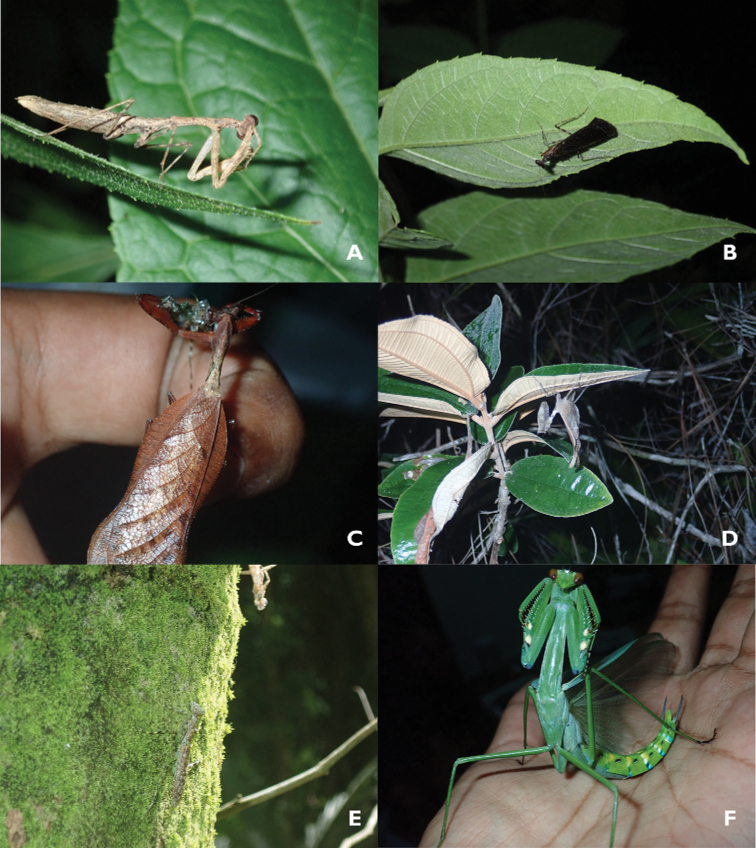
Acanthopoidea of Belize **A***Musoniolarapax* (Saussure & Zehntner) adult female from Las Cuevas Research Station **B***Pseudomiopteryxinfuscata* (Saussure & Zehntner) adult male from Las Cuevas Research Station **C, D***Acanthopsgodmani* (Saussure & Zehntner) adult male (**C**), adult female (**D**) from T.E.C. **E***Liturgusamaya* (Saussure & Zehntner) adult female next to exuvia at T.R.E.E.S. **F***Macromantisnicarague* (Saussure & Zehntner) adult male from Las Cuevas Research Station.

**Remarks.** See Fig. 2B.

##### SUBFAMILY THESPINAE Saussure, 1869

***Musoniolarapax* Saussure & Zehntner, 1894 (New country record) [SC**]

**Type locality.** Costa Rica: Caché.

**Material examined.** BZE: Stann Creek – 2 ♀ collected by hand, 1 ♂ collected by soap trap in the vicinity of Las Cuevas Research Station.

**Remarks.** See Fig. 2A.

***Thesprotia* sp. (New country record) [BD**]

**Type locality.** Species: *Thesprotiainfumata* (Serville, 1839) Brazil: Unknown.

**Material examined.** Belize: Belize Dist. 2 nymphs collected by hand in the vicinity of Tropical Education Center.

**Remarks.** Collected material were juveniles, making assigning sex and species designation difficult. As a result, we simply note the presence of the genus in the country.

#### FAMILY LITURGUSIDAE Giglio-Tos, 1915

***Liturgusamaya* Saussure & Zehntner, 1894 [BD, SC**]

= *Liturgusacharpentieri* Giglio-Tos, 1927

**Type locality.** Mexico: N. Yucatan, Temax.

**Material examined.**1 ♂, 2 ♀ collected by hand in the vicinity of Tropical Education Center, 1 ♀ collected by hand in the vicinity of T.R.E.E.S. Field Station.

**Remarks.** See Fig. 2E.

***Liturgusazoae* Svenson, 2014 [TD**]

**Type locality.** Guatemala: Alta V. Paz.

**Material examined.** None.

**Remarks.** Record from Svenson 2014.

#### FAMILY PHOTINAIDAE Giglio-Tos, 1915

##### SUBFAMILY MACROMANTINAE Brunner von Wattenwyl, 1893

***Macromantisnicarague* Saussure & Zehntner, 1894 (New country record) [CD, SC**]

= Macromantisovalifoliavar.nicarague Saussure & Zehntner, 1894

= *Macromantisnicarague*: Kirby 1904: 272; Giglio-Tos 1927: 324; Beier 1935: 121.

= *Macromantishyalina*: Rehn 1935: 212 (*partim*); Terra 1995: 73 (*partim*); Cerda 1997: 34 (*partim*); Salazar 1998: 108 (*partim*); Salazar 1999 (*partim*).

= *Macromantisovalifolianicarague*: Maes 1989: 16.

= *Macromantisnicarague*: Maes and Roy 1999: 63; Salazar 2000b: 74; 2002: 80.

**Type locality.** Nicaragua: W. Chontales.

**Material examined.** BZE: Cayo Dist: 3 ♂, 1 ♂ nymph, 1 ♀ nymph vicinity of Las Cuevas Research Station, BZE: Stann Creek Dist: 1 ♂ vicinity of Bocawina Rainforest Resort.

**Remarks.** See Fig. 2F.

#### FAMILY ACONTISTIDAE Giglio-Tos, 1905

***Acontistacordillerae* Saussure, 1869 (New country record) [SC**]

**Type locality.** French Guiana: St. Laurent du Maroni.

**Material examined.** BZE: Stann Creek Dist.: 1 ♀ collected by hand in the vicinity of Bocawina Rainforest Resort.

**Remarks.** This species is widely distributed in the Neotropics with records from Brazil to Mexico (Ehrmann 2002). Given records in nearby countries, there are likely other *Acontista* species in Belize, further sampling is required.

#### FAMILY ACANTHOPIDAE Burmeister, 1838

***Acanthopselegans* Lombardo & Ipollito, 2004 (New country record) [CD**]

**Type locality.** Guatemala: Morales.

**Material examined.** BZE: Cayo Dist.: 1 ♀ collected by hand in the vicinity of Las Cuevas Research Station.

***Acanthopsgodmani* Saussure & Zehntner, 1894 [BD**]

**Type locality.** Belize: unknown.

**Material examined.** BZE: Belize Dist.: 2 ♂ collected at light trap, 1 ♀ collected by hand in the vicinity of Tropical Education Center.

**Remarks.** See Fig. 2C, D.

***Pseudacanthopscaelebs* (Saussure, 1869) [SC, CD**]

**Type locality.** Mexico: Veracruz, Orizaba

**Material examined.** BZE: Stann Creek Dist.: 1 ♂ collected at light trap in the vicinity of Toucan Ridge Education and Ecology Society • BZE: Cayo Dist.: 5 ♂ collected at light trap in the vicinity of Las Cuevas Research Station.

#### FAMILY MANTIDAE Latreille, 1802

##### SUBFAMILY CHOERADODINAE Saussure, 1869

***Choeradodisrhombicollis* (Latreille, 1833) [SC, CD**]

= *Choeradodisperuviana* (Serville, 1839)

= *Choeradodisservillei* (Wood-Mason, 1880)

= *Choeradodisbrunneri* (Wood-Mason, 1882)

**Type locality.** Unknown (Roy 2004b).

**Material examined.** BZE: Stann Creek Dist.: 2 ♂ collected at light trap in the vicinity of T.R.E.E.S. Field Station • BZE: Cayo Dist.: 3 ♂ collected at light trap in the vicinity of Las Cuevas Research Station.

**Figure 3.**
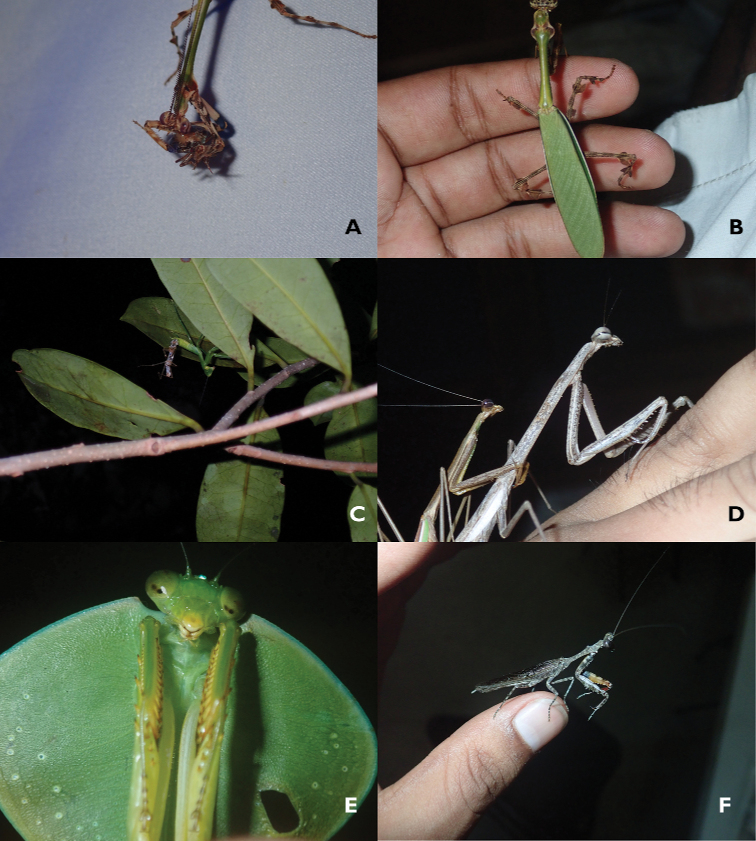
Mantidae of Belize **A***Vateschopardi* (Deeleman-Reinhold) adult male from T.E.C. **B***Vatespectinicornis* Stål adult female from T.R.E.E.S. **C***Stagmomantisfraterna* Saussure & Zehntner female nymph molting to subadult at T.E.C. **D***Phasmomantissumichrasti* (Saussure & Zehntner) adult pair mating from T.E.C. **E***Choeradodisrhombicollis* (Latreille) adult male from Las Cuevas Research Station **F***Mellieramordax* Rehn adult male from T.E.C.

**Remarks.** See Fig. 3E. Given the huge range of *Choeradodisrhombicollis* Latreille in Central and South America, it is more than likely that this species is present in all districts.

##### SUBFAMILY MELLIERINAE

***Mellieramordax* Rehn, 1935 (New country record) [BD**]

**Type locality.** Guatemala: Santa Emilia Pochuta.

**Material examined.** BZE: Belize Dist.: 4 ♂ collected at light trap, 2 ♂ nymph collected by hand in the vicinity of Tropical Education Center.

**Remarks.** See Fig. 3F. Females proved difficult to find, given this species’ morphology it is likely their behavior is similar to *Tarachodes* Burmeister or *Orthoderella* Giglio-Tos, in that individuals prostrate themselves onto a branch or twig.

##### SUBFAMILY STAGMOMANTINAE Brunner von Wattenwyl, 1893

***Stagmomantiscarolina* (Johansson, 1763) [BD**]

= *Stagmomantisirrorata* Johansson, 1763

= *Stagmomantisfuscata* Weber, 1801

= *Stagmomantisconspersa* Burmeister, 1838

= *Stagmomantisconspurcata* Serville, 1839

= *Stagmomantiscuticularis* Serville, 1839

= *Stagmomantisinquinata* Serville, 1839

= *Stagmomantisferox*, Saussure, 1859

= *Stagmomantisamericana* Taylor, 1862

= *Stagmomantiswheelerii* Thomas, 1875

= *Stagmomantisthoracica* Rehn, 1911

= *Stagmomantismaculosa* Chopard, 1912

= *Stagmomantisnordica* Giglio-Tos, 1917

= *Stagmomantispolita* Giglio-Tos, 1917

= *Stagmomantissimplex* Giglio-Tos, 1917

**Type locality.** USA: Carolina.

**Examined material.** BZE: Cayo District: 3 ♂ collected by hand in the vicinity of Tropical Education Center • BZE: Stann Creek Dist.: 1 ♂ collected at light trap in the vicinity of T.R.E.E.S Field Station.

***Stagmomantisfraterna* Saussure & Zehntner, 1894 [BD**]

= *Stagmomantismaya* Saussure & Zehntner, 1894

= *Stagmomantislatipennis* Beier, 1935

**Type locality.** Mexico: Tabasco, Teapa.

**Examined material.** BZE: Cayo District: 2 ♀ collected by hand, 3 ♂ collected by hand, 2 ♂ collected by light trap, in the vicinity of Tropical Education Center.

**Remarks.** See Fig. 3C.

***Stagmomantisheterogamia* Saussure & Zehntner, 1894 (New country record) [CD**]

**Type locality.** Panama: Bugaba.

**Examined material.** BZE: Stan Creek Dist.: 2 ♂ collected by hand in the vicinity of Las Cuevas Research Station.

***Stagmomantismontanamontana* Saussure & Zehntner, 1894**

= *Stagmomantisandrogyna* Saussure & Zehntner, 1894

= *Stagmomantistyphon* Rehn, 1904

= *Stagmomantiscintipes* Giglio-Tos, 1917

**Type locality.** Mexico: Guerrero, Chilpancingo, 4000 ft.

**Examined material.** None.

**Remarks.** Record added from Maxwell (2014).

***Stagmomantisvicina* Saussure, 1870**

= *Stagmomantiscentralis* Giglio-Tos, 1917

= *Stagmomantissimilis* Giglio-Tos, 1917

**Type locality.** South America: Unknown.

**Examined material.** None.

**Remarks.** Record added from Maxwell (2014).

***Phasmomantissumichrasti* (Saussure, 1861) (New country record) [BD, CD**]

= *Phasmomantismexicana* (Saussure, 1861)

**Type locality.** Mexico: Mexico Calida, Cordova.

**Material examined.** BZE: Belize Dist.: 2 ♂ collected by hand, 1 ♀ collected by hand, 1 oothecae collected by hand, in the vicinity of Tropical Education Center • BZE: Cayo Dist.: 3 ♂ collected by hand, 2 oothecae collected by hand in the vicinity of Las Cuevas Research Station.

**Figure 4.**
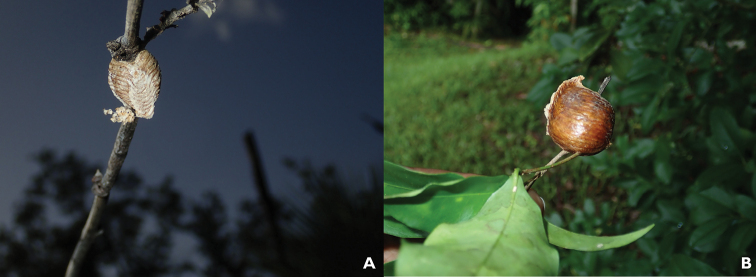
Oothecae of *Phasmomantissumichrasti* (Saussure, 1861), highlighting the morphological variation in different habitats **A** tropical savannah, and **B** tropical broadleaf rainforest. Oothecae from broadleaf rainforests were around twice as large as oothecae from tropical savannah and contained a hollow space between the eggs and outer wall.

**Remarks.** See Fig. 3D. In the rainforest populations, the morphology of the oothecae is greatly modified (Fig. 4). The oothecae are larger, have an air-filled space, and are much darker in color.

##### SUBFAMILY VATINAE Stål, 1877

**Tribe Vatini Stål, 1877**

***Vateschopardi* (Deelman-Reinhold, 1957) (New country record) [BD**]

**Type locality.** Mexico: Vera Cruz and Tabasco.

**Material examined.** BZE: Belize Dist.: 2 ♂ collected at light traps in the vicinity of Tropical Education Center.

**Remarks.** See Fig. 3A. This taxon is a possible synonym of *Vatespectinata* Saussure (Julio Rivera pers. comm). It is easily distinguished from *V.pectinicornis* Stål by the small lobe on anterodorsal margin of the forefemora and its presence only in tropical savannah habitat.

***Vatespectinicornis* (St**å**l, 1877) (New country record) [SC**]

**Type locality.** El Salvador: Unknown.

**Material examined.** BZE: Stann Creek Dist.: 3 ♂ collected at light traps, 4 ♀ collected at light traps in the vicinity of T.R.E.E.S. Field Station.

**Remarks.** See Fig. 3B.

### Dichotomous key to known Mantodea of Belize

Note: color characters are more accurately assessed for live individuals.

**Table:** 

1	Pronotum quadrate, small Mantodea, 30 mm or less, appearing to mimic wasps (adults) or ants (juveniles), promargin setose	***Mantoidamaya***
–	Pronotum not quadrate, longer than wide. Size and mimicry variable. Promargin with few setae or none	**2**
2	Cyclopean ear present. Appears as deep pit, located ventrally between mid and hind coxae. Forefemur with three or four posteroventral spines. Length variable, juxtaocular process not raised	**3 (Mantidae)**
–	Cyclopean ear absent (MSMT, DO forms). Forefemur with five or more posteroventral spines; if four or less, then species that are typically 50 mm or less in length, juxtaocular process typically raised past the posterior margin of the eye	**12 (Acanthopoidea)**
3	Lamellar margin of pronotum greatly expanded. Large, green mantids, mimicking leaves, with a conspicuous black marking on promargin of forefemur. Hindwing red at base	***Choeradodisrhombicollis***
–	Lamellar margin of pronotum not expanded. Size, color, and forefemora pattern variable	**4**
4	Ocellar process present, as bifurcated horn, length variable. Color typically shades of brown, mimicking twigs. Both sexes macropterous. Antennae in adult males pectinate, legs lobed	**5 (*Vates*)**
–	Ocellar process absent. Color green to gray, mimicking various colors of foliage. Females brachypterous, males macropterous. Antennae in both sexes filiform, legs lacking lobes	**6 (Stagmomantinae, Mellierinae)**
5	Forefemur with noticeable curve, lobe on anterodorsal margin of forefemur. Lateral margin of pronotum dentate only in anterior region of metazone	***Vateschopardi***
–	Forefemur only slightly curved. Forefemur without lobe on anterodorsal margin. Entire lateral margin of metazone dentate	***Vatespectinicornis***
6	Typically under 50 mm in length, stick and bark dwelling. Mid and hind femora pilose, with rows of long setae. Mid and hind limbs shorter than forelimbs. Gray wings, and patterned promargin of forelimbs. Metazone smooth, except for a conspicuous pair of tubercles present on anterodorsal region. Mottled in coloration, like bark	***Mellieramordax***
–	Size, habitat variable. Mid and hind femora not pilose. Mid and hind limbs longer or similarly sized to forelimbs. Wing coloration, forelimb patterning variable. Tubercles on anterodorsal region of metazone absent. Color variable	**7 (Stagmomantinae)**
7	Longer than 60 mm, elongate, males typically brown, with green anterior margin on forewing, female color variable. Males macropterous, females brachypterous with wings less than 15 mm. Eye spot present on hindwing as a smoky circle, four posteroventral spines	***Phasmomantissumichrasti***
–	Size, coloration, wing patterning and length variable. Lacking eyespots on hindwings, 3 or 4 posteroventral spines	**8 (*Stagmomantis*)**
8	Length 50 mm or less	**9**
–	Length greater than 50 mm (rarely below)	**10**
9	Body green, lacking colored stigma on forewings. Female hindwing yellow, tessellated, male hindwing hyaline with green veins. Prozone not concave. Two green or brown dots present on ventral margin of femoral promargin	***Stagmomantisfraterna***
–	Body color variable, lacking colored stigma on forewings. Female hindwing anteriorly yellow, posteriorly fuscus. Male hindwing with smoky tessellation in anal field, wing veins variable. Prozone concave. Two brown dots on ventral margin of the femoral promargin	***Stagmomantisvicina***
10	Black stigma present on forewing in both sexes. Color variable, size between 40–60 mm. Forefemur with two black dots.	***Stagmomantiscarolina***
–	Black stigma absent on forewings. Body color variable, body greater than 60 mm in length. Forefemur with or without black dots on ventrad of promargin	**11**
11	Stigma absent on forewing in both sexes. Bicolored, with brown on posterior region of the metazona. Hindwing with smoky with tessellation on the posterior margin in males. Females hyaline. Gracile species, promargin with smoky coloration and two black dots	***Stagmomantisheterogamia***
–	Coloration variable. Male hindwings typically hyaline (though tessellation rarely present on some individuals in the median of the anal field). Females with yellow tessellated hind wings and white pterostigma. Moderately robust species, promargin lacking smoky coloration and black dots	***Stagmomantismontanamontana***
12	Typically slender mantises, foretibial spination variable. Sometimes with multiple apical spines, especially when nymphs. Antennomeres with setae longer than the length of each antennomere in adult males. Females apterous, males winged. Often mimicking sticks (when not, body robust and length less than 30 mm), habitat variable	**13 (Thespidae)**
–	Habitats, body shape, spination, and wings variable, not as above	**15**
13	Body slim, foretibia less than 1/3 the length of forefemur, foretibia with multiple apical spines. Low hanging species, appearing grass-like	***Thesprotia***
–	Body, foretibia, habits variable	**14**
14	Length 50 mm or less, with many granulations and tubercles on pronotum. Slim species, abdomen with parallel lateral margins. Forefemora curved towards apex, abdomen with short lobes medially on the posterior margin of tergites, foretibia with multiple apical spines. Lacking ocellar process	***Musoniolarapax***
–	Length 50 mm or less, with a granulated pronotum approximately diamond shaped. Stout species with rounded lateral abdominal margins. Forefemora stout, lacking apical curve. Abdomen with short lobes medially on posterior margin of tergites, foretibia with one apical spine. Ocellar process, appearing as a small horn, arising just behind median ocellus	***Pseudomiopteryxinfuscata***
15	Five or more posteroventral spines. Eyes pointed or rounded. When pointed, ocular projection present. When rounded, dorsal margin of eyes slightly below vertex of the head. Wings in females either modified for camouflage, or brightly patterned. Cranial processes present, lost in some taxa	**16 (Acanthopoidea: Acontistidae, Acanthopidae)**
–	5 or more posteroventral spines. Eyes rounded, ocular projection absent. Lacking ocellar processes. Dorsal margin of eyes at or below vertex of the head. Cranial processes absent, size variable	**19 (Acanthopoidea: Liturgusidae, Photinaidae)**
16	Five posteroventral spines. Typically 30 mm or less. Nymphs appear to mimic *Pseudomyrmex* spp (Formicidae: Pseudomyrmecinae), adults are colored similarly to inflorescences (e.g., orange, green, red, white). Process on vertex of cranium absent	***Acontistacordillerae***
–	Six posteroventral spines. Coloration typically browns, blacks, though some white and green may be present; size 30 mm or larger. Nymphs and adult females mimic leaves, lichen, moss, or twigs. Adult males mimic dead leaves. Wings in females modified for camouflage, appearing as dead leaves. Ocular processes present in all taxa, length of process variable. Process on vertex of cranium present or not	**17 Acanthopidae**
17	For males (females unknown): forefemora covered with multiple tubercles. Short, quadrate vertical process. Abdominal sternites flanged. Forefemora with lobe near forecoxal-forefemoral margin in both sexes. Pronotum with a pair of large, black circles near posterolateral margin of metazone. Adults mimic dead leaves (females unknown)	***Pseudacanthopscaelebs***
–	Males and females with or without tubercles (when present, found on outer face of the forefemur, dorsal face of the pronotum, gena, post gena). Vertical process absent. Abdominal sternites flanged. Forefemora lacking lobe near forecoxal-femoral margin. Pronotum without a pair of large, black circles near posterolateral margin of metazone. Mimicking dead leaves in both sexes	**18 (*Acanthops*)**
18	Slimmer, more gracile species, occipital area with two small tubercles (one per eye) when viewed slightly posteroventrally	***Acanthopsgodmani***
–	Robust species, occipital area lacking such tubercles in males, flattened tubercle present in females (one per eye)	***Acanthopselegans***
19	Large, typically exceeding 90 mm. Tergites colored with blue and white markings. Forefemora with white eyespots basally, and black markings on forefemoral-forecoxal margin. Often found below leaves with arms held below and slightly out to the side. Supraanal plate elongate, triangular	***Macromantisnicarague***
–	Small, typically less than 50 mm. Body dorsoventrally flattened. Tergites and forefemora variable. Supraanal plate enlarged and rounded at apex. Found on bark. Extremely quick mantises. Sulcus between first and second posteroventral femoral spine, to receive enlarged second tibial spine	**20 (*Liturgusa*)**
20	Lamellar margins strongly concave, with a short lamellar expansion, which is flattened dorsoventrally. Posterior margin angled approximately 45 degrees from center of body	***Liturgusazoae***
–	Lateral margins slightly concave, lacking lateral expansion. Posterior margin quadrate	***Liturgusamaya***

### Biogeographical remarks

Belize has several types of tropical habitats. The northern states of Belize are lowland tropical savannah and mixed tropical forest, whereas the southern states are at higher elevations, due to the Maya Mountain range. The mountain range passes through parts of the Cayo, Stann Creek, and Toledo districts. These districts are dominated by tropical broadleaf rainforest, regardless of elevation, though urbanization has created other types of habitats. The largest diversity of species, as expected, was found in tropical broadleaf rainforests.

Microhabitat preferences of Mantodea are largely unknown. As a result, though previous studies have mentioned habitats where various taxa have been found (Rivera and Svenson 2016), details such as foliage preference and level (i.e., understory, canopy) are still being ascertained for many species. Noting these findings is important to understanding speciation and life histories of various taxa. For example, Svenson (2014) highlighted the diversity of Liturgusidae in various levels of the rainforest foliage. Such findings necessitate that future studies include further collection details, so more data can be gathered regarding habitat information, and possibly uncover hidden diversity. We include below some observational notes for the genera collected.

#### Acanthops

Only one species was previously known in Belize, *A.godmani* (Lombardo and Ippolito 2004; Patel and Singh 2016a, 2016b). Given that the species is found in tropical and subtropical habitats, it is no surprise that it was collected in the drier T.E.C. *Acanthopselegans* was seemingly restricted to tropical broadleaf rainforests and was described from specimens from the tropical rainforests of Guatemala and Costa Rica by Lombardo and Ippolito (2004). One adult female and one nymph were collected at Las Cuevas Field Station, on low hanging tree branches (<3 m off the ground).

#### Choeradodisrhombicollis

Given that *C.rhombicollis* is found from the Western Andes to southern Mexico, it seems to be restricted to broadleaf rainforests. Sightings of individuals from tropical mixed forests were reported from T.E.C. by the employees of the site. However, given the confusion of insects by citizen scientists and lack of physical specimens, we do not officially note its presence at this location. This species can be found in Mexico in a few non-rainforest habitats, as recorded in iNaturalist. These individuals tend to be smaller with a less conspicuous pronotum, possibly indicating foliage as a selecting force on morphology. Males were collected at lights from T.R.E.E.S and Las Cuevas Research Station. Individuals were seemingly absent from Bocawina; however, this is likely due to inadequate sampling rather than the species’ absence.

#### Mantoida

This genus is represented by one species. Populations of *Mantoidamaya* showed noticeable variation in size and coloration across the different habitats where they were occurred. In T.E.C., individuals were several millimeters smaller and had a black and white coloration. However, in Las Cuevas, individuals (when alive) were blue as well. It appears that adults may mimic in color pattern and behavior some ichneumonid wasps (see subtribe Mesostenina). The observed variation in size and coloration could be based on selective pressures to better mimic the local wasp species. Mantoida were very common and were found typically on branches 2–3 m above ground at night. Individuals readily flew to lights and headlamps.

#### Musoniola

*Musoniolarapax* is the only species in this genus that we collected and included only two adult females and one adult male. The adult male was collected from a light trap low to the ground, but the females were collected on low foliage and on the ground at Las Cuevas Field Station, possibly indicating a lifestyle as forest floor hunting insects.

#### Phasmomantis

Seemingly only one species of *Phasmomantis* was collected in Belize, *P.sumichrasti*. Unexpectedly, *P.sumichrasti* oothecae displayed morphological differences based on habitat. We were able to confirm the two oothecae were of the same species. From the population at T.E.C., an adult female of *P.sumichrasti* laid an oothecae in captivity, which we were able to match with wild-collected oothecae. For the population from Las Cuevas Research Station, we were able to find images from Belizean citizen science pages of adult females laying matching oothecae (https://www.facebook.com/groups/1425989061003132, Creatures of Belize). The wild-collected oothecae both hatched during the trip, and the first instar nymphs were seemingly identical. While first instar morphology is fairly homologous within genera, we feel that the evidence found and examination of genitalia from adult males confirm that these two populations are of the same species.

*Phasmomantissumichrasti* did not show much preference for any habitat and was found in tropical broadleaf forest, tropical savannah, and mixed tropical forest. However, all individuals were collected relatively close to the ground (<1.5 m). The differences in oothecae morphology are highlighted below in Figure 4. In the broadleaf forest at Las Cuevas Research Station, oothecae had a hollow space between the outer layer and the eggs, within which we found nesting *Camponotusplanatus* Roger, 1863 in both oothecae collected. Given that *C.planatus* is a generalist in terms of nest sites, it is possible that the rainforest populations developed this varied structure in response to environmental conditions, and the ants opportunistically took advantage of this space to nest. Unexpectedly, the ants did not consume the eggs. Further research is needed to determine whether this is a type of defensive mutualism, as the ants readily emerged from the oothecae to defend their nest when disturbed.

Only adult males were collected in Las Cuevas Research Station, but these individuals were around 5–10 mm longer than individuals collected in T.E.C. and had a slightly larger eyespot on their hind wings. Males were darker in color. The genitalia examined exhibited only minor morphological differences. The combination of these differences suggests a possible early case of speciation, which will need to be further examined.

#### Pseudacanthopscaelebs

This species’ distribution was similar to *Choeradodisrhombicollis* in Belize. Multiple males were collected at lights in both T.R.E.E.S. and Las Cuevas Field station. Females have not been collected and are undescribed, leaving microhabitat preference unknown for both sexes. Females of other species are noted to hang upside down from mossy branches in the wild (Lombardo et al. 2013). It is not surprising that only males, which are readily found at light traps, were collected. Given the crypsis of this genus, nymphs and adult females are rarely collected, as they are difficult to locate visually and are not attracted to light traps.

#### Pseudomiopteryxinfuscata

*Pseudomiopteryxinfuscata* was commonly encountered and found in nearly every sampled location. Adult females were only collected at T.E.C. and Bocawina, but males were found at all other locations. Most males were perched on or underneath foliage at the edge of the forest at night. It seems elevation does not affect the distribution of the species, as this species has been recorded from multiple locations in Central America at varying elevations and on various types of foliage, indicating the adaptability of this species.

#### Stagmomantis

The genus *Stagmomantis* shows incredible morphological and ecological diversity (Maxwell 2014), and their distribution in any given habitat is also varied. *Stagmomantisfraterna* was found frequently at T.E.C. and was found anywhere from 2–5 m above the ground on small shrubs, trees, and other foliage on and under leaves. *Stagmomantiscarolina* was also common at T.E.C. and was found 2–3 m off the ground on various shrubs and trees. *Stagmomantiscarolina* tended to stick more closely to the stems and trunks of the foliage and was found in both T.E.C. and T.R.E.E.S. *Stagmomantisheterogamia* was collected only at Las Cuevas Field Station, atop large, broadleaf plants, 5–8 m above the ground, and were found only when pulling the plants down to view the tops of the leaves.

#### Vates

We found both species of *Vates* at lowland locations. The genus was absent at Las Cuevas Research Station (elev. 650 m), although more sampling will have to be done in order to rule out their presence at higher elevation sites. Almost all individuals were found at lights in T.E.C. and T.R.E.E.S., making it difficult to glean information on microhabitat preference. *Vateschopardi* was collected in T.E.C., whereas *Vatespectinicornis* was collected in T.R.E.E.S. Given that *V.chopardi* is found in Mexico, it appears to be restricted to arid and subtropical zones. Svenson et al. (2016) noted the possible synonymy between *V.pectinata* and *V.chopardi*, which is plausible given their overlapping distribution and morphological similarity. Neither of these species has been previously recorded from Belize, however. They are speculated to be a canopy dwelling group (Julio Rivera pers. comm.)

#### Possible other species

The following are species that have not been found, but possibly occur in Belize, based on records (Ehrmann 2002; Patel and Singh 2016a, 2016b; Patel et al. 2016) in nearby countries (Mexico, Honduras, Guatemala): *Angelachampioni* Saussure & Zehntner, 1894, *Angelamiranda* Saussure, 1871, *Hondurantemnachespiritoi*Rodrigues et al., 2017, *Oligonyxbicornis* Saussure & Zehntner, 1894, *Oligonyxbidens* Saussure & Zehntner, 1894, *Oligonyxdorhnianus* Saussure & Zehntner, 1894, *Oligonyxmaya* Saussure & Zehntner, 1894, *Oligonicellabolliana* Saussure & Zehntner, 1894, *Oligonicellapunctulata* Saussure & Zehntner, 1894, *Oligonicellascudderi* Saussure, 1870, *Oligonicellastriolata* Saussure & Zehntner, 1894, *Oligonicellatessellata* Saussure & Zehntner, 1894, *Pseudovateschlorophea* Blanchard, 1836, *Pseudovatescornuta* Saussure & Zehntner, 1894, *Pseudovateslongicollis* Stål, 1877, *Stagmomantislimbata* Hahn, 1835, *Yersinia* Saussure, 1869. Further sampling is required to determine the presence of these 17 taxa.

## Conclusion

We expand our knowledge of Belizean mantis fauna by adding 12 new species, bringing the total known species in Belize to 21. The newly found diversity of mantis fauna in Belize highlights the importance to spend more time sampling more countries in Central America to better understand the distribution and diversity of Mantodean fauna. Understanding these distributions can help quantify natural variation as a result of vicariance by distance.

We can also gain valuable information on the ecology of the taxa we found, and how this influences morphology in certain populations. The ability for a species to vary morphologically in different habitats has been demonstrated in several studies in Mantodea (Lombardo and Ippolito 2014; Svenson 2014; Svenson et al. 2016), suggesting that the morphology in Mantodea is highly plastic, and can vary in response to environmental selective pressures.

Given the recent discovery of taxa such as *Metacanthops* Agudelo & Maldaner, 2019 and *Hondurantemna*Rodrigues et al., 2017 it is not inconceivable that more cryptic and/or monotypic genera remain undiscovered in areas previously sampled. The habitats across Central America vary quite drastically even in the same country, and the presence of several smaller mountain ranges, such as the Maya Mountains, can lead to speciation by allopatry as these ranges can serve as reservoirs or new habitats for existing taxa to colonize, or relicts to persist in.
